# Induced Negative Mood Increases Dictator Game Giving

**DOI:** 10.3389/fpsyg.2018.01542

**Published:** 2018-08-21

**Authors:** Carolina Pérez-Dueñas, M. Fernanda Rivas, Olusegun A. Oyediran, Francisco García-Torres

**Affiliations:** ^1^Department of Psychology, Maimónides Biomedical Research Institute of Córdoba, Reina Sofía University Hospital of Córdoba, University of Córdoba, Córdoba, Spain; ^2^Middle East Technical University – Northern Cyprus Campus, Güzelyurt, Turkey; ^3^Department of Economics, University of Castilla-La Mancha, Albacete, Spain

**Keywords:** negative mood, stress, decision making, dictator game, altruism, emotions

## Abstract

The study examines the influence of induced negative mood on dictator game giving (DGG) with two recipients. Participants (*N* = 63) played the role of a dictator in a three-player dictator game. They could choose among two options: an altruistic option, where two receivers receive 10 Euros and the dictator himself receives nothing, or a selfish option, where the dictator himself receives 5 Euros and both receivers receive nothing. For half of the participants, the second option entailed that only one receiver receives nothing and the other receives 10 Euros. After four rounds, participants were randomly assigned to look at 10 pictures with either positive or negative emotional content with the purpose of inducing positive or negative mood. The results show that looking at pictures with negative emotional content increases anxiety and skin conductance and increases DGG in the remaining four rounds of the game. On the other hand, whether the selfish option would imply that one or both recipients receive nothing does not seem to have a strong influence on DGG.

PsycINFO Classification code: 2340; 2360.

## Introduction

The dictator game (DG) is an economic game in which one person is assigned the role of the dictator, who determines an allocation of some endowment (e.g., a sum of money) between himself and one or more other players, and often in a completely anonymous setting with zero opportunity for reciprocal punishment. As suggested by the classical economic theories, the allocation decision by a rational decision maker tends to self-maximize the benefits as much as possible. However, empirical evidence suggests that dictators often give between 30 and 50% of the pie to others instead of simply maximizing their own benefit (Forgas, 2016).

It is important to note that framing affects dictator game giving (DGG). Accordingly, a meta-study shows that dictators share significantly more in games with multiple recipients and when there is a high social link between the dictator and the recipient (Engel, 2011).

Related to the affective states or mood induced to the dictators, studies have shown that DGG is sensitive to such manipulations. Thus, authors as Capra (2004) and Ibanez et al. (2017) showed that positive mood increases DGG, while Tan and Forgas (2010) found that DGG is increased by negative mood. In the study of Ibanez et al. (2017) and Tan and Forgas (2010), the participants had to decide how much money they wanted to send to the recipient, whereas in the study of Capra (2004) participants chose between three alternatives.

The purpose of this study is to contribute to the literature about the effect of induced mood on DGG with two recipients, when dictators have to choose between two options: a purely altruistic option and a purely selfish option.

## Materials and Methods

### Participants

The experiment involved 189 students from the University of Granada, Spain. Sixty-three of the subjects who were primarily non-economics students acted as dictators, while the remaining 126 subjects who played the passive roles as recipients were economics students who were recruited while participating in an Experimental Economics course during which their individual pictures were taken. Dictators were randomly assigned to two groups: a *Negative Stimuli group* (*NS*; 10 males and 22 females) and a *Positive Stimuli group* (*PS*; 11 males and 20 females).

### Materials and Procedure

Each of the 63 dictators was seated comfortably in a quiet room at a distance of about 60 cm from the computer screen used for the experiment and privacy during the experimental process was guaranteed.

The participants played eight rounds of a game on economic decision making. In each round, each dictator *S* was matched with different pairs of recipients (*Q*&*R*) such that each dictator *S* saw a total of eight different pairs of recipients. Each pair was randomly selected from the pool of 126 recipients, and the pair was never repeated again in the eight rounds played by each dictator *S*.

The dictators had to choose between an altruistic or a selfish option by pressing a key.

The choice instructions were as follows: “You can choose between (a) giving 10 Euros to each of the players Q&R and keeping 0 Euros for yourself [10, 10, 0], or (b) giving 0 Euros to each of the players Q&R and keeping 5 Euros for yourself [0, 0, 5]”.

For half of the participants (31), the conditions of the selfish option were modified as: “You can choose between (a) giving 10 Euros to each of the players Q&R and keeping 0 Euros for yourself [10, 10, 0], or (b) giving 0 Euros to player Q, 10 Euros to player R, and keeping 5 Euros for yourself [0, 10, 5]”.

We include this manipulation to explore if giving money to one recipient in the selfish option is strong enough to change the level of DGG (although the other receiver receives nothing).

After the first four rounds, the mood-induction started. To induce negative mood to the *NS* group, a set of 10 pictures from the International Affective Picture System (IAPS; Lang et al., 2005) with negative emotional content with mean valence and arousal values of 1.79 (SD = 1.3) and 7.47 (SD = 2), respectively, were presented accompanied by brief unpleasant texts that emphasized uncertainty and lack of control. To induce positive mood to the *PS* group, a set of 10 pictures from the IAPS with positive emotional content with mean valence and arousal values of 7.77 (SD = 1.50) and 4.41 (SD = 2.53), respectively, were presented accompanied by brief pleasant texts that emphasized joyful moods about life. This mood induction procedure has been previously used to induce high and low state anxiety (see Pacheco-Unguetti et al., 2010; Pérez-Dueñas et al., 2014)^1^.

At the end of the eight rounds, the participants had to randomly draw a piece of paper from a plastic bag containing eight folded pieces of paper each with one number (from 1 to 8) corresponding to each round played in the game. The selected round was used to calculate the payoffs of the three players. The dictators were paid before leaving the laboratory, while the passive recipients received their individual payments at a later date.

With the purpose of checking the effectiveness of the mood induction, the dictators fill in the Spanish version of the Spielberger State Anxiety Scale (STAI-state; Spielberger et al., 1994) before round 1 and before round 5, and the dictators’ skin conductance was recorded during the experiment^2^.

The whole procedure lasted about 1 h. **Table 1** shows the decision schedule.

**Table 1:** The decision schedule in the eight DG rounds.

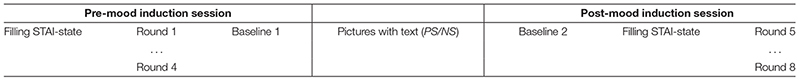

## Results

### Effect of the Affective Manipulation

Paired-samples *t*-tests show that for the *NS* group, the mean level of anxiety measured by the STAI-state is higher in the post-mood induction session of the experiment (mean: 25.09; SD: 12.44) than in the pre-mood induction session (mean: 14.34; SD: 7.76) [*t*(31) = -6.02, *p* = 0.001, *d* = -1.06]. For the *PS* group, the mean level of anxiety is lower in the post-mood induction session (mean: 12.61; SD: 7.21) than in the pre-mood induction session (mean: 15.35; SD: 6.98) [*t*(30) = 2.18, *p* = 0.038, *d* = 0.39]. Paired-samples *t*-tests show that for the *NS* group, the mean level of skin conductance is higher in the post-mood induction session (mean: 4.05; SD: 2.48) than in the pre-mood induction session (mean: 3.56; SD: 2.44) [*t*(31) = -3.330, *p* = 0.002, *d* = -0.59]. However, the level of skin conductance for the *PS* group was not statistically different between the post-mood induction session (mean: 3.28; SD: 1.40) than in the pre-mood induction session (mean: 3.39; SD: 1.80) [*t*(30) = 0.713, *p* = 0.482, *d* = 0.13].

These tests show that while the positive stimuli reduce anxiety levels (as measured by STAI) and have no effect on the skin conductance, the negative stimuli increase anxiety levels and skin conductance.

### Effect of Emotional Induction on DGG

In the first half of the experiment before being exposed to the positive or negative stimuli, the subjects chose the altruistic option 1.30 out of 4 times (32.54%) on average. In the second half of the experiment after exposure to the stimuli, the subjects selected the altruistic option 2.30 out of 4 times (57.5%), but with a large difference relative to the group.

**FIGURE 1:**
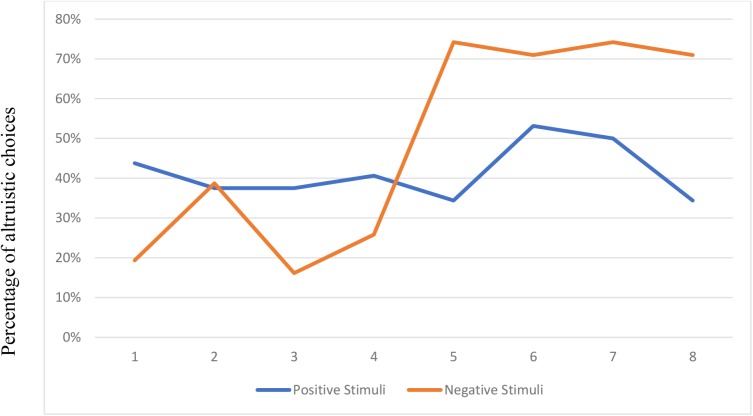
Percentage of altruistic choices by round and type of stimuli.

**Figure 1** shows the percentage of altruistic choices in each round for the subjects exposed to the positive stimuli and the negative stimuli^3^.

**Figure 1** clearly shows that there is an important effect of the type of stimuli. While the percentage of altruistic choices does not vary much when the subjects are exposed to the positive stimuli, the percentage dramatically changes when they watch the negative stimuli. The effect is immediate and does not wear off (at least in the four rounds the subjects play): it goes from 26% in round 4 to 74% in round 5 and it stays above 70% in rounds 6, 7, and 8.

To corroborate this effect, we ran several *t*-tests. The matched-samples *t*-tests show that the average number of times the altruistic option was chosen in the pre- and post-mood induction does not differ for the subjects exposed to the positive stimuli [*PS*; pre-mood induction: 1.65 (1.36); post-mood induction: 1.77 (1.48)] [*t*(30) = -0.36, *p* = 0.719, *d* = -0.07], but is significantly different for the subjects exposed to the negative stimuli [*NS*; pre-mood induction: 0.97 (1.12); post-mood induction: 2.81 (1.53)] [*t*(31) = -5.21, *p* = 0.001, *d* = -0.92].

In a mixed ANOVA we find a strong effect of the interaction between time (pre-induction vs. post-induction) and type of stimuli [*F*(1,61) = 11.68, *p* = 0.001, *d* = 0.88]. Independent samples *t*-tests show a difference in favor of the *PS* group prior to the viewing of images, that is, in the first part of the experiment, the altruistic option was chosen more frequently in the *PS* group than in the *NS* group [*t*(61) = 2.16, *p* = 0.035, *d* = 0.55]. On the other hand, the difference is in the other direction after viewing the images [*t*(61) = -2.74, *p* = 0.008, *d* = -0.69]. This change is explained by the fact that while the average number of times the altruistic option was chosen by the *PS* group does not change significantly between the first and second parts of the experiment, the opposite occurs in the *NS* group as the subjects’ altruism notably increases in the second part. The fact that in the first part of the experiment, the subjects in the *PS* condition chose the altruistic option more frequently was unexpected, but this makes the effect of the negative mood induction even stronger.

## Discussion

This research indicates that negative mood induction significantly increases DGG with two recipients when the dictator has to choose between an altruistic and a selfish option. Our results are in line with recent findings regarding the effect of negative mood induction on prosocial behavior where participants have to decide whether to donate or share an amount of money with other people (Tan and Forgas, 2010; Dickert et al., 2011; Verhaert and Van den Poe, 2011; von Dawans et al., 2012; Margittai et al., 2015; Steinbeis et al., 2015; Sollberger et al., 2016). In these studies, participants experiencing negative mood, had to decide how much of their endowment they would give to the receiver/s. However, this is the first study that has investigated how negative mood induction influenced dictators who had to choose between a purely selfish option and a purely altruistic option.

Similarly, cognitive load increases altruism in a DG when the dictators have to decide between two options (e.g., Schulz et al., 2014). Although the load was not manipulated in our study, the self-reported anxiety and arousal were increased in the *NS* group and it is well known in the literature that anxiety affects cognitive resources (e.g., Eysenck et al., 2007). Many studies in the past showing the link between load and prosociality confounded altruism and norm-following behavior, because the dictator had to choose between a selfish option and 50/50 split norm (e.g., Rand et al., 2012). However, our findings suggest that the effect even goes beyond the standard norm: even when there is no normative option, negative mood pushes decision makers towards the prosocial option. The drawback is that this difference in the design makes our results not directly comparable with those from other DG papers.

Finally, there were no differences in DGG when the selfish option implied that the dictator was the only player out of the three who earned money or when two of the three players earned money (one of whom was the dictator). However, due to the small sample, we cannot say that it does not influence it at all but it seems that it is not a very powerful determinant of DGG. Future studies must be conducted to explore this issue further.

Despite limitations, the current study provides initial evidence that people who are induced toward a negative mood increases DGG even when being altruistic means that they get no money.

## Ethics Statement

This study was carried out in accordance with the recommendations of Psychology Department of the University of Granada. The protocol was approved by the Psychology Department of the University of Granada. All subjects gave written informed consent in accordance with the Declaration of Helsinki.

## Author Contributions

The manuscript has been seen and reviewed by all authors (CP-D, MR, OO, and FG-T) and all authors have contributed to it in a meaninful way.

## Conflict of Interest Statement

The authors declare that the research was conducted in the absence of any commercial or financial relationships that could be construed as a potential conflict of interest.

## Footnotes

1 Detailed information on the emotional stimuli and database can be found in https://osf.io/2dk5f/?view_only=8738e03a70ec40d08249775261247b2e and https://osf.io/cek2h/?view_only=7a750aa278fb4fde87ec117b2f09667e

2 Skin conductance was obtained using 6-mm inner diameter Ag/AgCl electrodes (TSD203 connected to the GSR100C amplifier) filled with electrode gel (GEL101). Electrodes were placed on the middle phalanx of the index and middle finger of the left hand according to the recommendations of Fowles et al. (1981). Skin conductance was recorded using the Biopac MP150 system and Acq*Knowledge* software 3.9.1.6 on a PC running under Windows XP at a rate of 125 Hz in continuous mode. To measure the baseline skin conductance, the subjects were asked to remain quiet and calm for 5 min while fixating on a dot in the middle of the screen before and after the stimuli presentation (Baseline 1 and Baseline 2). The experiment was run on a computer with a 1 GHz Pentium III processor connected to a 15-inch VGA monitor. E-prime software was used for randomly displaying the recipients’ photographs to the dictators, the mood-induction process, and for recording the choices of each dictator (Schneider et al., 2002).

3 As there are no differences between participants which were presented the selfish option with one (soft selfish—SS) or both recipients receiving nothing (hard selfish—HS), we carry out the analysis with both groups together. There are no treatment effect on the average number of times the altruistic option is chosen in pre-mood induction: *t*(61) = -0.72, *p* = 0.250, *d* = -0.18 [*SS*: 1.19 (1.18); *HS*: 1.42 (1.39)] and post-mood induction: *t*(61) = -1.72, *p* = 0.090, *d* = -0.43 [*SS*: 1.97 (1.66); *HS*: 2.65 (1.45)]. Moreover, the matched-samples *t*-tests show that altruism was lower in part 1 (pre-mood induction session) than in part 2 (post-mood induction session) in both treatments [*SS*: *t*(31) = -2.15, *p* = 0.040, *d* = -0.38; *HS*: *t*(30) = -3.02, *p* = 0.005, *d* = -0.54].
